# The synergy between natural polyphenol-inspired catechol moieties and plant protein-derived bio-adhesive enhances the wet bonding strength

**DOI:** 10.1038/s41598-017-10007-8

**Published:** 2017-08-29

**Authors:** Zhong Wang, Shujun Zhao, Ruyuan Song, Wei Zhang, Shifeng Zhang, Jianzhang Li

**Affiliations:** 1 MOE Key Laboratory of Wooden Material Science and Application, Beijing Forestry University, Beijing, 100083 China; 2 Beijing Key Laboratory of Wood Science and Engineering, Beijing Forestry University, Beijing, 100083 China

## Abstract

Novel soybean meal-based biomimetic (STP) adhesives were fabricated via soybean meal (SM) and enhanced by tannic acid (TA) and polyetheylenimine (PEI) (TAPI) co-crosslinking network based on natural polyphenol-inspired chemistry. The multiple physico-chemical interactions (including intermolecular H-bonding and covalent bonding) between the TAPI co-crosslinking system and SM matrices were examined by the Fourier transform infrared spectroscopy, solid-state ^13^C nuclear magnetic resonance, X-ray diffraction, thermogravimetric analysis, and scanning electron microscopy. The results showed that a dense, robust, and water-resistant adhesive layer was constructed between network-bound catechol moieties in the TAPI and SM system, endowing the STP ﻿adhesive﻿ with high wet bonding strength for plywood. As expected, TAPI-modified SM adhesives showed a 156.1% increase in wet bonding strength compared to the control SM adhesive. The adhesion meets standard requirements for interior-use plywood. Both the solid content and residual mass analysis also confirmed that the enhancement in the STP adhesive was attributable to the network crosslinking density and stiffness after integrating the TAPI system. Moreover, the thermal stability of the resultant STP adhesive exhibited a significant improvement. The proposed STP adhesive may be a promising cost-effective and wet-resistant bio-adhesive for the application in the wood composites industry.

## Introduction

In recent studies, bio-based adhesives have shown remarkable potential in manufacturing industry applications due to their numerous advantages in terms of low cost, biodegradability, renewability, and environmental friendliness in comparison to petroleum-based adhesive materials^1–3^. Typically, the fabrication of wood composites using non-formaldehyde bio-adhesives necessitates enhanced bonding strength; this is typically accomplished by modifying the biomass matrices^4, 5^.

As a notable example^6, 7^, soybean-derived bio-adhesives generally exhibit low adhesion strength and low water resistance due to their poor chemical resistance and inherent amphiphilic nature^8–11^. The formation of cross-linking network has been acknowledged as an effective approach to fabricate high-adhesion soy protein-based adhesives by incorporating reactive cross-linkers and synthetic resins per their high reactivity with the amino (–NH_2_), carboxyl (–COOH), and other exposed groups of polypeptide side chains^12^. Cross-linking modification includes the use of formaldehyde-based resin^13^, maleic anhydride^14^, epoxy groups^15^, epoxy resin, and monomer (e.g., ethylene glycol diglycidyl ether and 1,2,3-propanetriol-diglycidyl-ether)^10, 16^. These reaction conditions, cost, and safety of organic compounds are unfavorable, however^17^.

Recently, many researchers have been inspired to explore catechol-containing molecules by the robust bioadhesive ability of mussel adhesive proteins (MAPs)^18, 19^. The mussel-inspired artificial adhesive-based catecholamine chemistry has been extensively developed to facilitate strong and reversible adhesion under various “extreme” (e.g., humid, underwater, or with polar substrate surface) conditions^20^. Generally, available methodologies can be roughly sorted to three categories: 1) designed or functionalized synthetic or natural polymers with 3,4-dihydroxyphenyl-L-alanine (DOPA) or catechol-based monomers produced via organic/polymer synthesis technologies^21, 22^, 2) the direct conversion of protein tyrosine residues to DOPA-o-quinones via Fremy’s salt oxidation^23^, and 3) the replication and production of MAPs in bio-systems via the technique of genetic engineering^24^. Mussel-inspired adhesives are designed based on a bio-polymer backbone including chitosan^25^, gelatin^22^, hyaluronic acid^26^, and vegetable protein^27^. They exhibit favourable adhesion capability with suitable biocompatibility. For example, Li *et al*. introduced DOPA groups to the backbone of soy protein isolate to mimic mussels as a biomass-based adhesive^28^; the copolymer exhibited strong bonding strength and water resistance relative to unmodified soy protein adhesive. It is well recognized that MAPs-mimetic approaches are dominated by multifunctional catecholamine monomers like DOAP^26^, hydrocaffeic acid^25^, 3,4-dihydroxybenzaldehyde^21^, and norepinephrine^29^. Unfortunately, the methods that necessitate catecholamine monomers are relatively complicated or expensive in cost, which seriously limits their practical application (especially for the industrial application)^30^.

Natural polyphenol derivatives, which possess diverse chelation and adhesion abilities, have recently emerged as a notable alternative to catecholamine monomers^31, 32^. Most phenolic compounds (e.g., caffeic, ferulic, and gallic acids as well as epicatechin) have been shown to construct a mechanically strong protein-based films through intermolecular interactions with proteins^33^. The intra/intermolecular H-bond is weak resistant to water corrosion in adhesive materials, however. Small molecules of phenolic compounds in the cross-linking system are not robust enough to yield desirable wet-resistant bio-adhesive properties. Lee *et al*. reported an effective medical adhesive comprised of catechol/pyrogallol-rich tannic acid (TA) formulated with functionalized multi-arm poly(ethylene glycol) (PEG) that can be formulated via physical blending^34^. The mucoadhesive properties of the adhesive are a result of strong intermolecular H-bonds between a functionalized multi-arm PEG and TA. In a previous study conducted in our laboratory, it was found that caffeic acid and tri-functional aziridine crosslinking networks could yield stable, robust composite films with favorable mechanical properties^35^. Actually, TA can undergo versatile reactions with –SH/–NH_2_ terminated molecules due to the chemical multiple functionality, which endowed the stronger interactions with amino-containing polymer^36, 37^. Compared with other amino-terminated polymers such as functionalized multi-arm PEG, poly(ethyleneimine) (PEI) is a hydrophilic and low-cost amino-rich polymer, generally can react with TA through Michael addition and/or Schiff-base formation between amine and catechol^38, 39^. Therefore, the co-crosslinking of TA with amino-rich PEI and natural protein matrices represents a promising solution for constructing multi-interactive and robust network structures for wet-resistant bio-adhesives.

This paper proposed a facile and “green and cheap” modification technology for fabricating soybean meal-based biomimetic (STP) adhesives via the co-crosslinking of a TA/PEI (TAPI) system within a soybean meal (SM) matrices based on natural polyphenol-inspired chemistry. As shown in scheme 1, the low-cost and amino-rich PEI was chosen as a skeleton to frame the branched network plus network-bound catechol moieties in TAPI as a potential crosslinker interacting with –NH_2_ groups of the soy protein peptide chains to form multiple physico-chemical interactions (including intermolecular H- bonds and covalent bonds) and to promote the robust cross-linking of the STP system. After further optimizing the TAPI concentration in the STP adhesive, the chemical structures, thermal and rheological properties, and morphologies of the resultant modified bio-adhesives were investigated to evaluate their adhesion strength and water resistance. The as-prepared biomass-based bio-adhesives were comprised of readily available components, low in cost, and suited to large-scale production in the wood-based composite industry.

## Results and Discussion

**Figure 1:**
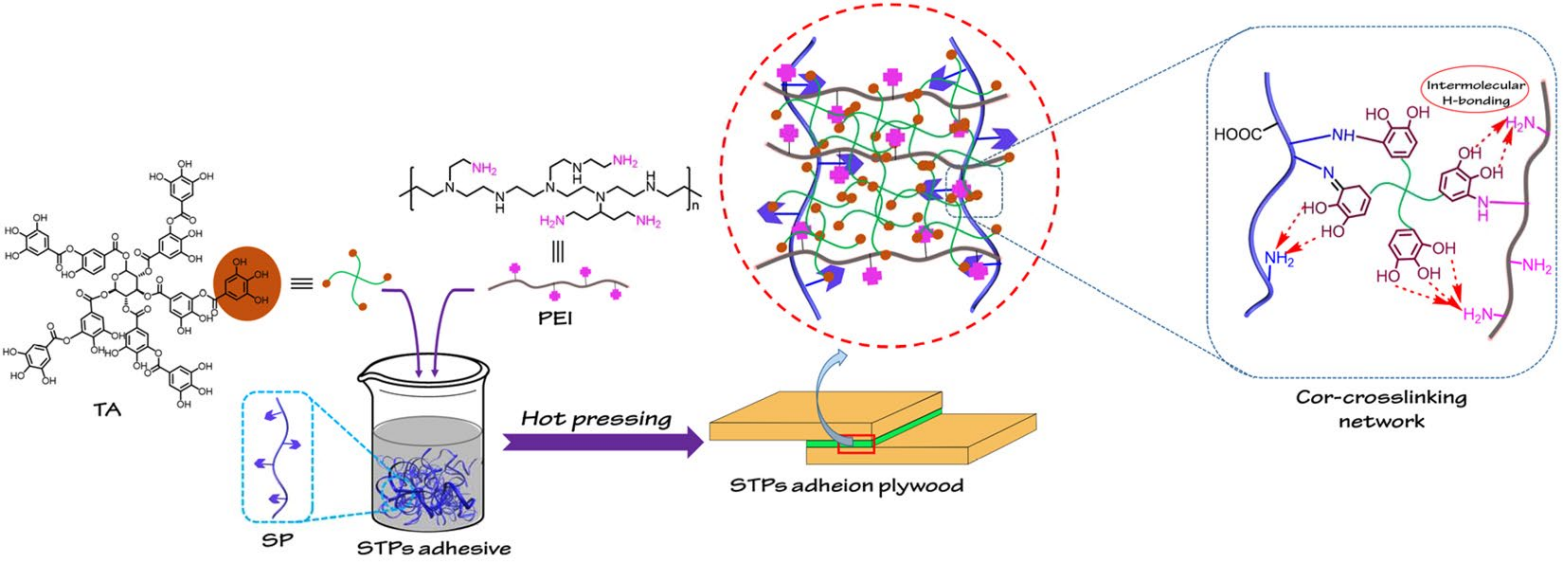
Schematic representation of preparation and crosslinking formation of STP adhesive.

In this study, a series of natural polyphenol-inspired STP adhesives was fabricated via SM and natural polyphenol-inspired TAPI co-crosslinking system. As shown in Fig. 1, the network-bound catechol groups in TA readily undergo auto-oxidation in basic conditions and the quinone and/or catechol groups reacted with PEI and soy protein via covalent bonds (Michael-type addition or Shiff base reaction)^9, 40^, which were also capable of forming stronger intermolecular H-bonds in the reaction system. The multiple physico-chemical interactions between TAPI and SM resulted in the formation of a robust crosslinking network.

### Solids content and dynamic viscoelastic analysis

Solids content is one of the most important physical parameters of a given adhesive in terms of its practical application in plywood bonding due to its impact on performance during the curing process^38^. The SC of the various adhesives are listed in Table 1. The unmodified SM (control) adhesive showed the lowest SC at 27.66%, representing a flow issue^39^. The SC of the adhesives was strongly dependent on the TA concentration and PEI addition. After 20 wt% PEI or TA were incorporated, the SC of the adhesives increased gradually to 32.34% and 31.54%, respectively. Compared to the control, the SC of the STP-10, STP-20, and STP-30 adhesives increased to 33.6%, 40.34%, and 43.66%, respectively.

**Table 1:** SC and RM of various SM-based adhesives: (A) SM, (B) STP-10, (C) STP-20, (D) STP-30, (E) SP-10, (F) SP-20, (G) ST-10, (H) ST-20.

Entry	A	B	C	D	E	F	G	H
Solid content (%)	27.66	33.6	40.34	43.66	28.8	32.34	30.14	31.54
Residual mass (%)	75.26	79.50	81.78	82.39	70.13	75.03	80.86	80.50

**Figure 2:**
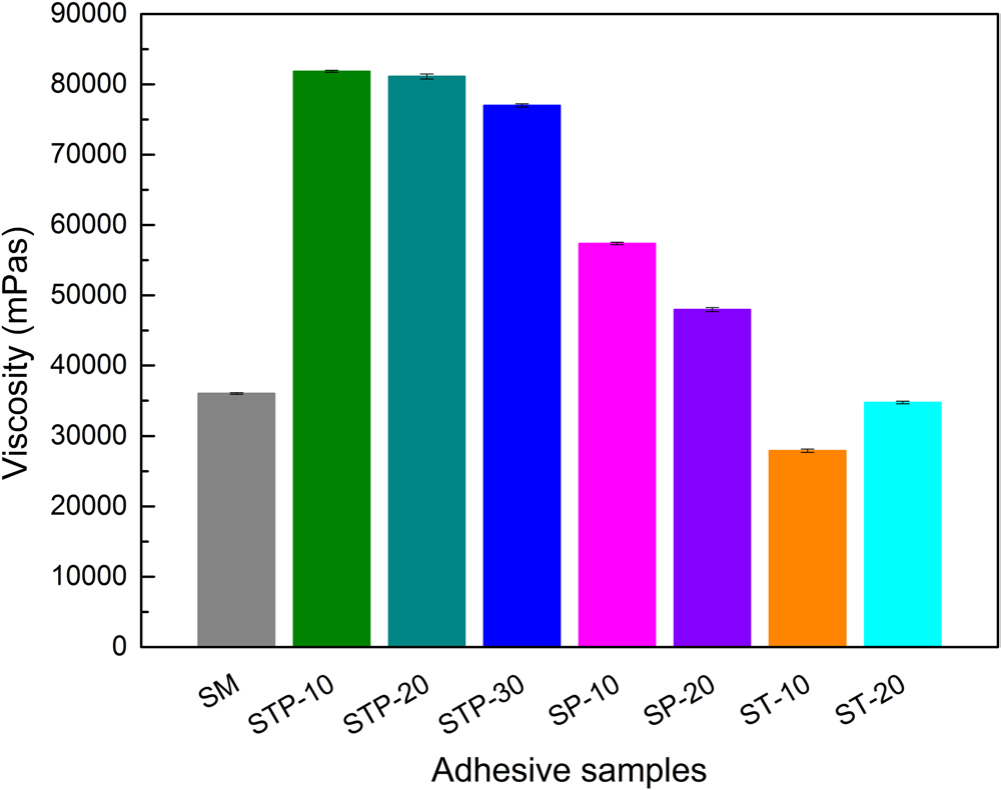
Initial viscosity of various SM-based adhesives.

Viscosity influences the handling of adhesives during the practical application. In generally, an appropriate viscosity range (5 000–25 000 mPa*·*s) is required to ensure good flow-ability of adhesives in wood composites. The initial apparent viscosities of the various adhesives are shown in Fig. 2. The modified STP adhesives had higher viscosity, which firstly increased from 36 030 to 81 850 mPa*·*s then declined slightly as the TAPI addition increased; this indicated a distinct intermolecular interaction between TAPT and the SM matrices^34^. Though these STP samples with TAPI were very viscous and not well flow-able, the favorable softness still ensure it can be brushed evenly on veneer surface^4^. The viscosity of the modified adhesives dropped significantly after the same addition of PEI and TA, however, mainly due to poor interactions between the single PEI or TA and the SM matrices and reduced intermolecular forces in the soy protein molecules^41^. It was noticed that the co-crosslinking modification by integrating TAPI system into the SM matrix resulted in more notable effects on the adhesion strength compared to that of high viscosity.

### Structure and reactivity analysis

**Figure 3:**
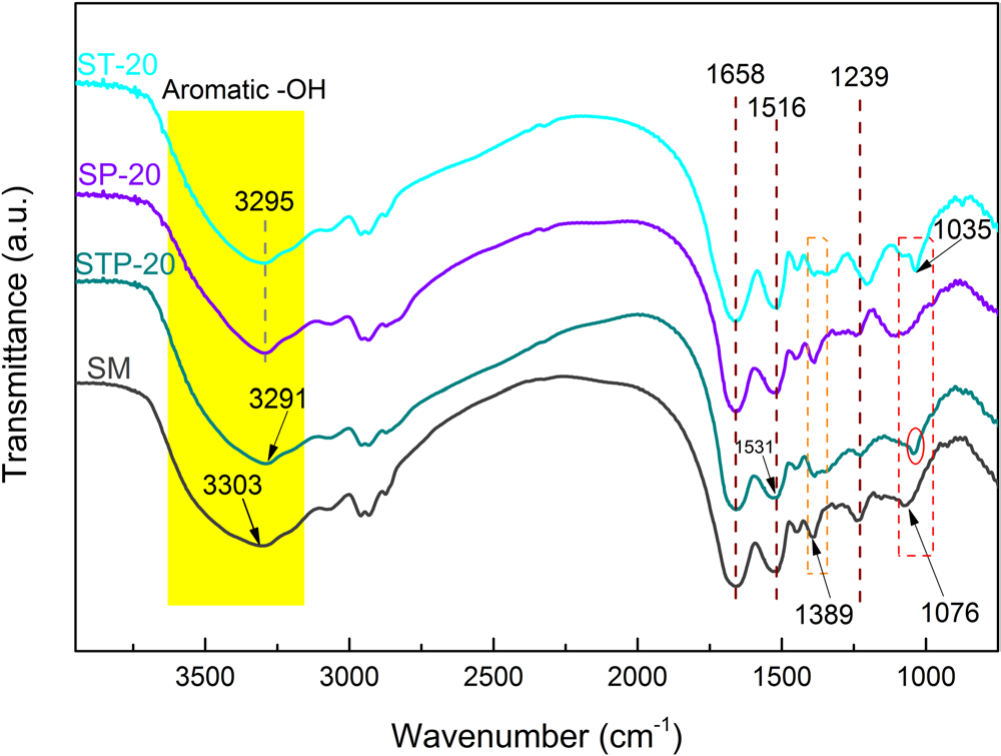
FTIR spectra of the various STP adhesives: SM, STP-20, SP-20, and ST-20 adhesives.

The FTIR characterization of STP adhesives was performed to confirm the presence of strong interactions in the ternary system as shown in Fig. 3. The typical amide bands at 1658, 1516, and 1239 cm^−1^ referred to amide I (C=O stretching), amide II (N−H bending), and amide III (C−N and N−H stretching) of the protein peptide, respectively^38^. The broad and strong absorption band at 3303 cm^−1^ can be attributed to free/unbound O–H and N–H stretching vibrations, and the peaks at 1389 and 1076 cm^−1^ to COO– and C–O bending vibrations, respectively^9, 16^. After the STP formulation, the O–H and N–H stretching peak of the STP adhesive shifted to a lower wavenumber (3291 cm^−1^), and the absorption band (3600–3100 cm^−1^) became broader, indicating that the catechol/pyrogallol −OH in TA probably participated in the adhesive system via intermolecular H-bonds^41, 42^. The amide I peak shifted from 1516 to 1531 cm^−1^ after integrating the TAPI co-crosslinking system, indicating effective physical and/or chemical interactions between TAPI and SM matrices^34^.

The vibration peak around 1389 cm^−1^ (COO– bending) became significantly weaker in the SM and STP-20 adhesive, indicating weakened vibrational energy of COO– bonding; this was probably caused by interactions with the catechol/pyrogallol −OH^41^. The C−O bending in STP-20 also shifted from 1076 cm^−1^ (SM) to 1043 cm^−1^ while the peak of ST-20 shifted to a lower wavenumber, 1035 cm^−1^. These changes in the absorption bands altogether indicated that the strong intermolecular interaction occurred between the TAPI and protein chains via multiple chemical and/or physical combinations.

**Figure 4:**
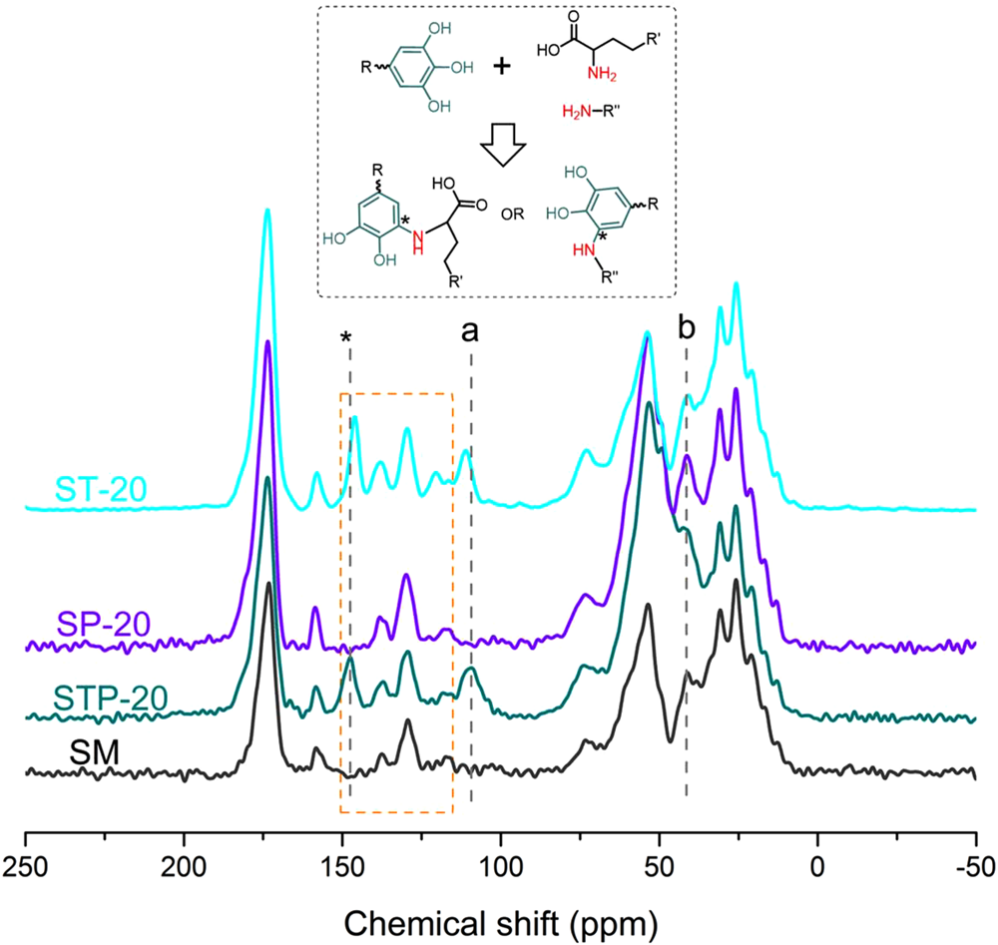
Solid-state ^13^C NMR spectrum of SM, STP-20, SP-20, and ST-20 adhesives.

Solid state ^13^C NMR spectroscopy was also applied to further investigate the chemical structure of STP (Fig. 4). The spectral features of protein peptide (Fig. 4a) were consistent with those reported by Ma *et al*. and Kang *et al*.^9, 43^: Major peaks corresponding to carbonyl (155–175 ppm), aromatic (115–130 ppm), *α*-carbon (45–65 ppm), *β*-carbon (25–45 ppm), and methylene and methyl groups (15–25 ppm) were observed far away from the backbone. Compared to those of the unmodified SM adhesive, the peaks for STP-20 in the range of 115–150 ppm became significantly broader, demonstrating that the stronger intermolecular H-bonds played an important role in STP formation^34^. The resonance of phenolic C-OH carbons from catechol/pyrogallol moiety in TA was observed at 146.0 ppm in the ST-20 adhesive^44^ and it was shifted to 147.5 ppm the STP-20 adhesive, suggesting that the change might be caused by the formation of C-N linkages between PEI and/or soy protein, and TA^45^. The characteristic peak at around 111.0 ppm shifted up-field in the STP-20 adhesive (Fig. 4a). Moreover, the resonance at 41.3 ppm could be attributed to aliphatic CH_2_ groups of SP-20 adhesive, while the peak distinctly became weaker for STP-20 adhesive, indicating that the co-crosslinking process changed the original PEI structure^46^. These results could be explained by that the mobility of C from SPI matrix was changed by the chemical cross-linking of TA with other PEI molecules or SPI chains to form linkage (Fig. 4), which was also confirmed the FTIR result.

**Figure 5:**
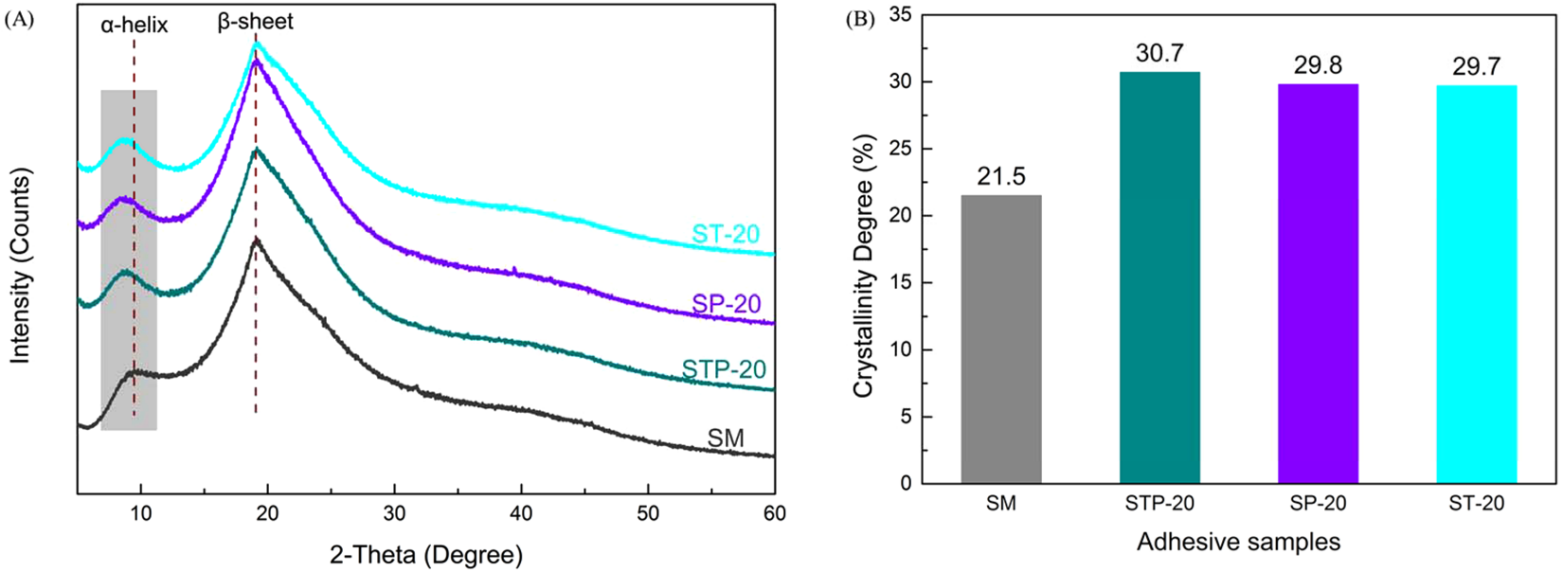
(**A**) XRD patterns and **(B)** crystallinity of SM, STP-20, SP-20, and ST-20 adhesives.

Corresponding to the aforementioned FTIR test findings, the TAPI co-crosslinking system was assumed to generate strong intermolecular interactions with the soy protein chains via H-bonds and covalent bonding, thus offering favorable cross-linking networks in the STP adhesive. The effects of the TAPI on the STP adhesives’ structure conformations were characterized by XRD (Fig. 5A). The broad characteristic peaks at 2*θ* = 9.6° and 19.3° in the SM adhesive correspond to the *α*-helix and *β*-sheet structures of soy protein secondary conformation, respectively, as reported by Kang *et al*.^9^. Compared to the control, the characteristic peak (*α*-helix) shifted from 9.6° to 8.6° and raised the intensity after TAPI incorporation resulting from the partial denaturation of protein molecules under the TAPI co-crosslinking system^35^. In addition, the crystallinity of resultant adhesives was calculated by XRD measurement. As shown in Fig. 5B, with the introduction of TAPI, TA, or PEI, the adhesive crystallinity increased to 30.7% and reached its maximum (STP-20 adhesive) compared to that of the unmodified SM adhesive. Generally, it is acknowledged that the crystallization is the ordered array of molecules and cross-linking will lead to its crystallinity reduction for the adhesive system^7^. However, this result clearly indicated that the crystallinity in STP adhesives was not associated with the usual cross-linking modification, but developed with the formation of amorphous network for SPI chains^47^. Most importantly, with the integration of TAPI, SPI segments were favorably re-arranged closely and orderly together, and more crystalline domain (including the crystallization of TAPI itself) interpenetrated into the STP adhesives^41^. Collectively, these changes in the structure conformations of adhesives demonstrated that the strong intermolecular interactions between TAPI and SM matrices were well identified as the dominant co-crosslinking network due to the active network-bound catechol moieties.

### Wet bonding strength

**Figure 6:**
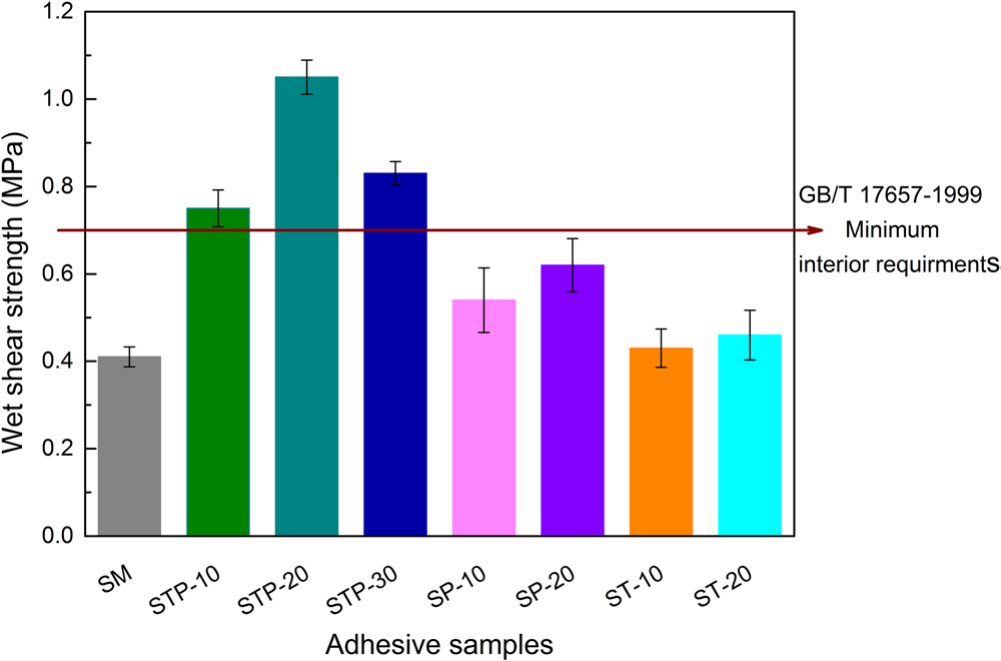
Tensile shear strength of plywood with different SM-based adhesives after immersion in water (63 ± 3 °C) for 3 h.

The wet bonding strength of the STP-bonded plywood significantly increased compared to the control ones, as shown in Fig. 6. The addition of TA or PEI had little influence on wet shear strength; all the bonding strength values failed to meet GB/T 17657–1999 requirements for interior-use plywood, likely due to the lack of effective intermolecular interaction and low solid content^16^. A significant improvement in wet bonding strength was realized with the incorporation of the TAPI co-crosslinking system compared to that of the adhesive system fabricated by adding the same TA or PEI content.

The wet bonding strength increased from 0.41 MPa to 1.05 MPa as the TAPI addition increased from 0 to 20 wt%, counting an increase of 156.1% compared to the plywood bonded with unmodified SM adhesive. The wet shear strength decreased to 0.83 MPa when TAPI addition was further raised to 30 wt%, however. To this effect, the strong intermolecular interactions between network-bound catechol groups in TAPI and the protein chains endowed the adhesives with high wet adhesion strength, while the muti-reaction sites further promoted the formation of a robust cross-linking network in the STP adhesive. The excessive addition of TAPI reduced the wet bonding strength of the plywood bonded with STP-30 adhesive, most likely due to the competition among surplus NH_2_ groups in PEI and peptide side amino groups with the catechol moieties as well as the native hydrophilicity of PEI chains^35^. This phenomenon indicated that the reinforcement effect of TAPI was closely related to the appropriate reaction ratio of TAPI with the SF matrices. The TAPI co-crosslinking combination could be effective in significantly reinforcing the wet adhesion performance of STP adhesives.

### Insoluble analysis

The effects of adding PEI, TA, or TAPI on the residual mass (RM) of the cured adhesives after the hydrolyzation were examined as presented in Table 1. The RM increased when different amounts of TA or TAPI were incorporated. Nevertheless, only PEI addition reduced the RM (SP-10 and SP-20 adhesives) as hydrophilic PEI could not bond effectively with the peptide chains, resulting in the dissolution of small molecular substances in the cured adhesive^48^. The TAPI co-crosslinking system underwent effective physical and/or chemical combination with the peptide chains, as confirmed by FT-IR and solid-state ^13^C NMR results, which increased the RM of the cured adhesive. After TAPI was incorporated, the RM of the STP adhesives increased by 5.6% (STP-10), 8.7% (STP-20), and 9.5% (STP-30), respectively, compared to that of the unmodified SM adhesive. These results further confirmed that the strong intermolecular interactions in the STP adhesives promoted robust cross-linking of the peptide chains and formed dense polymeric networks, thereby limiting permeation by water molecules^39^.

### Thermal behavior analysis

**Figure 7:**
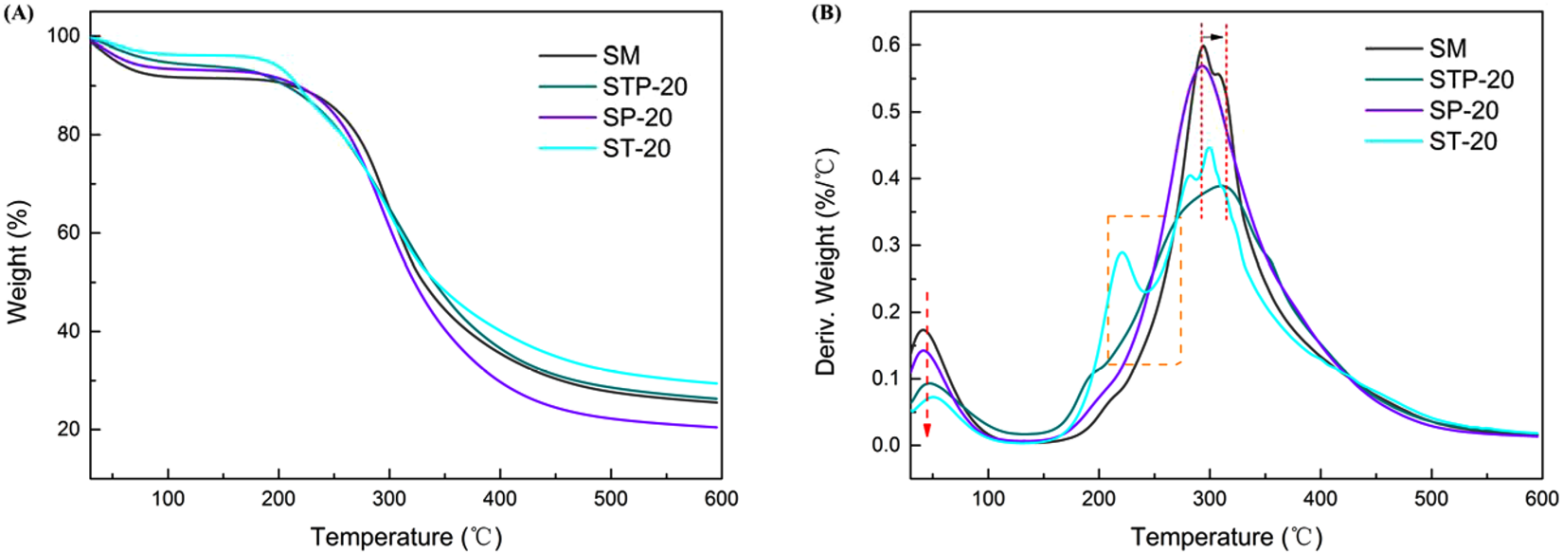
(**A**) TG and **(B)** DTG curves of SM, STP-20, SP-20, and ST-20 adhesives.

The thermal properties of the biomimetic STP adhesives were examined by TGA (Fig. 7); the thermo-degradation data were summarized and are listed in Table 2. The STP adhesives underwent a two-stage thermal degradation process. The initial weight loss from 30 °C to 150 °C occurred due to a dehydration reaction during the curing process, while the second stage within 150 °C to 450 °C was due to peptide backbone breakage^4, 9^. During this stage, the weight loss was probably resulted from the non-covalent bonds degradation, including intermolecular and/or intramolecular H-bonds and electrostatic bonds, hydrophobic interaction, and as well as the covalent bond cleavage of C−C, C−O, and C−N linkages in the peptide backbone^39^. The degradation peaks in the first stage (around 38 °C) of all the modified adhesives significantly decreased compared to the SM adhesive, where the peak temperatures at the maximum degradation rate (T_max_) shifted backward (Fig. 7B). These significant changes were primarily due to the subsequent thermal reaction of the adhesive system.

**Table 2:** Thermo-degradation data for SM, STP-20, SP-20, and ST-20 adhesives.

Entry	Peak1 (°C)		Peak2 (°C)		Peak3 (°C)	
	T_i_	T_max_	T_i_	T_max_	T_i_	T_max_
SM	36.78	42.77	257.21	294.10	—	—
STP-20	38.56	49.63	237.52	310.36	—	—
SP-20	37.01	43.81	248.15	293.07	—	—
ST-20	41.09	54.05	197.73	217.85	279.47	300.93

T_i_: Initial degradation temperature.

T_max_: Temperature of maximum degradation rate.

After incorporating TAPI in the adhesive formulation, the T_max_ values in the protein backbone degradation stage increased from 294.10 °C (SM) to 310.36 °C (STP-20) and exhibited a lower degradation rate compared to the control (Table 2), indicating the thermal stability enhanced in the STP adhesives. The degradation peak around 217.85 °C of the ST-20 adhesive was not retained in the STP-20 biomimetic adhesive, likely reflecting reactions among TA, PEI, and protein chains^4, 35^. The evident distinction in thermal behavior caused by the TAPI co-crosslinking system was mainly attributable to breakage in the strong intermolecular interactions between network-bound catechol groups and SM matrices, which caused the robust cross-linking network formation and increased the thermal stability observed. These results also revealed that the multiple interactions effectively disrupted the protein conformation after forming stable covalent bonds and intermolecular H-bonds, in accordance with the XRD results.

### Characterizing STP adhesives morphology

**Figure 8:**
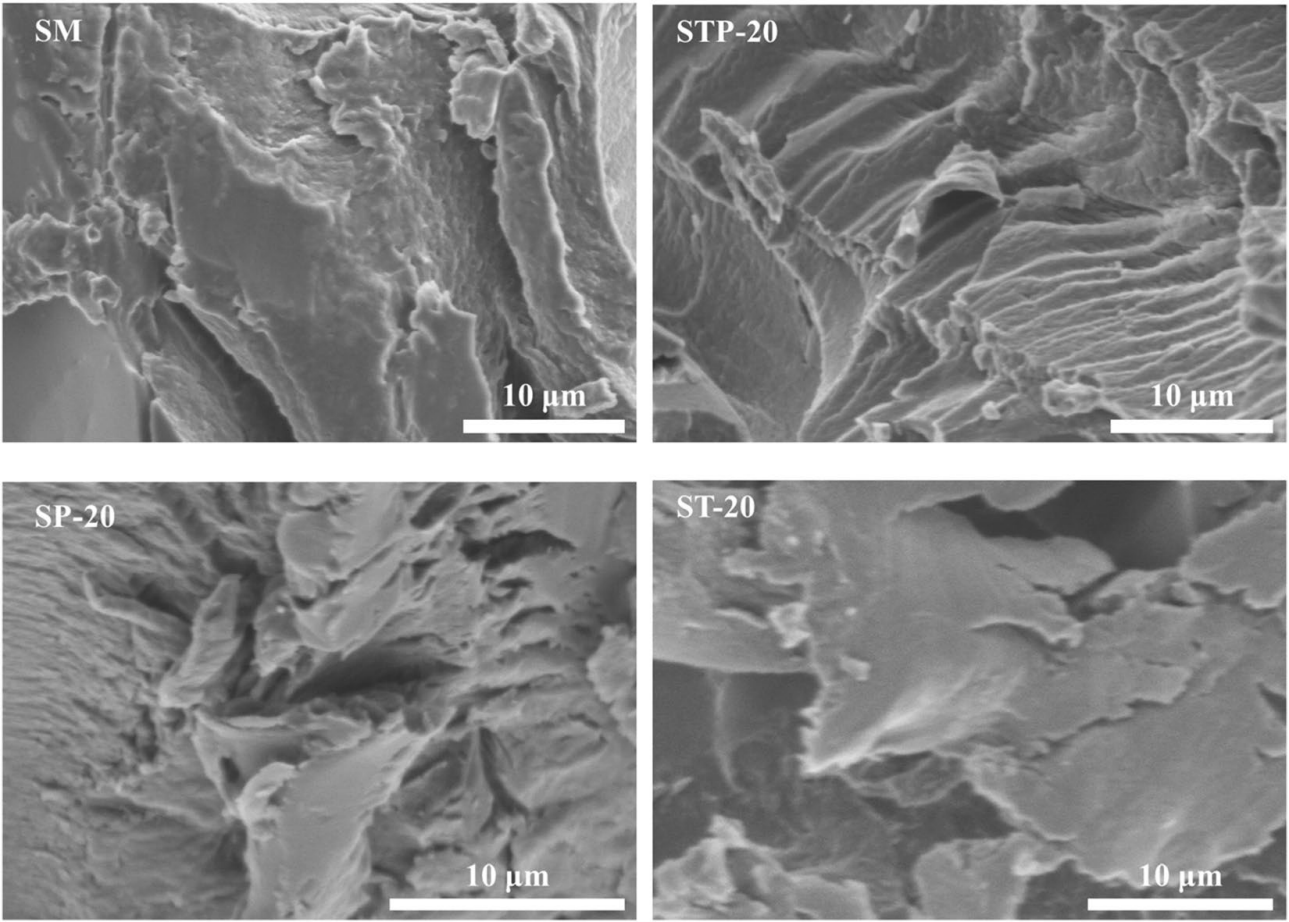
Fracture surface micrograph of various cured SM, STP-20, SP-20, and ST-20 adhesives.

The fracture surfaces of the cured STP adhesives were compared by SEM. Figure 8 shows that the unmodified SM adhesive exhibited a relatively loose and discontinuous fracture surface with some grooves and micropits, indicating the material’s inherent brittle and cleavage fracture^10, 35^. After the TAPI integration, the surface cracks of the control were shaved away and the fracture surface of the STP-20 adhesive became more compact, likely being a result of network-bound catechol moieties in TAPI physically and/or chemically combining with the SM matrices (per the FT-IR and solid-state ^13^C NMR analysis results). Figure 8 shows a surface morphology with intermittent cracks and large aggregations on the SP-20 and ST-20 adhesives, which occurred because the alone TA or PEI failed to combine effectively with soy protein chains. Overall, the strong intermolecular interactions in STP adhesives appeared to promote the cross-linking of the SP chains and to form robust polymeric networks. To this effect, they can be utilized to prepare high-performance bio-adhesives for wood composites.

## Conclusions

In this study, STP adhesives composed of TAPI and SM within a co-crosslinking network were prepared. This natural polyphenol-inspired STP adhesive exhibited enhanced water resistance compared to the unmodified SM-based adhesive. The network-bound catechol moieties in TAPI were combined with cross-link SM to enhance the intermolecular interactions between TAPI and the SM matrices. The physico-chemical interactions between TAPI and SM were confirmed by FT-IR, solid-state ^13^C NMR, and XRD analyses; SEM images verified the rearrangement of the soy protein chains and effective intermolecular cross-linkage after the TAPI integration.

The wet bonding strength of STP adhesive increased from 0.41 to 1.05 MPa after the modification while its SC and RM increased by 45.8 and 8.7%, respectively, compared to the control. The thermal stability of the modified STP adhesive was also improved. To this effect, it was believed that the results represented a facile, effective, bio-inspired method for physico-chemically combining plant polyphenol-derived TAPI with natural protein matrices that was readily applicable as an alternative to petroleum-based materials for developing environmentally friendly, cost-effective, and wet-resistant bio-adhesives.

## Materials and Methods

### Materials

The soybean meal (SM) powder (46% soy protein content) was purchased from Xiangchi Grain and Oil Company (Shandong, China) and milled to 200 mesh in a laboratory grinder. The tannic acid (TA, 95% pure) and poly(ethyleneimine) (PEI, Mw = 70000, 50 wt% aqueous solution) were obtained from Tianjin Heowns Biochem Co., Ltd. (China) and Aladdin (China), respectively. The Poplar veneer with 1.5 cm thickness and 40 × 40 cm^2^ nominal dimension (8% moisture content) was supplied by Wen’an (Hebei, China). Distilled (DI) water was used in all aqueous solution preparations.

### Preparation of Soybean Meal-based Biomimetic Adhesives

The procedure was operated according to our previously reported method as depicted in Scheme 1^16^. Briefly, the SM (28 g) was dispersed in DI water (72 g) under vigorous mechanical stirring for 30 min at 20 °C. A certain amount of TA and/or PEI (as listed in Table 3) was then added sequentially under constant stirring for 10 min at 20 °C to develop the soybean meal-based biomimetic adhesives. The control (unmodified) soy soybean meal adhesive was prepared under the same conditions but without the addition of TA or PEI. The formulated STP samples are described in Table 1.

**Table 3:** Experimental formulations and summary of various SM-based adhesives.

Adhesive codes	Soy flour (g)	Distilled water (g)	TA (g)	PEI (50 wt%, g)
SM	28.0	72	—	—
STP-10	28.0	72	2.8	2.8
STP-20	28.0	72	5.6	5.6
STP-30	28.0	72	8.4	8.4
SP-10	28.0	72	—	2.8
SP-20	28.0	72	—	5.6
ST-10	28.0	72	2.8	—
ST-20	28.0	72	5.6	—

### Preparation of Triple-layered Plywood Specimens

The adhesive was used to prepare three-ply wood specimens by coating each layer at spread rate of 180 g/m^2^. The hot-pressing temperature, pressure, and time were set to 120 °C, 1.0 MPa, and 315 s, respectively. After the hot-pressing, the dried plywood was stored in ambient conditions (25 ± 2 °C, 60 ± 5% relative humidity (RH)) for a period of 8 h before further testing.

### Wet Bonding Strength Measurements

The water resistance bonding strength of plywood was determined according to China National Standard GB/T 9846.3–2004 for type II plywood and methods described by Li *et al*.^38^. The test was carried out with three pre-treatments on the plywood specimens: 1) Twelve plywood specimens (25 mm × 25 mm) were cut from each plywood panels, 2) immersed in water (63 ± 3 °C) for 3 h, and 3) cooled to room temperature for 10 min before testing. The test was performed using the universal material testing machine (WDW-200E, Jinan, China) with a cross-head speed of 10 mm/min. The wet shear strength was calculated as follows:

1 $${\rm{Wet}}\,{\rm{shear}}\,{\rm{strength}}\,({\rm{MPa}})=\,\frac{{\rm{Force}}\,({\rm{N}})}{{\rm{Gluing}}\,{\rm{area}}\,({{\rm{mm}}}^{2})}$$

where Force is the tensile strength and Gluing area is a constant (625 mm^2^).

### Solid Content Analyses

The solid content (SC) of the adhesives was determined by weighing the difference in weight before (*m*_i_) and after drying (*m*_d_) in triplicate as follows:

2 $$\mathrm{SC}\,( \% )=[1-\frac{{m}_{i}-{m}_{d}}{{m}_{i}}]\times 100$$

where m_i_ is the initial weight of adhesive and m_d_ is the oven dry weight of the adhesive.

### Dynamic Viscoelastic Analyses

The apparent viscosity of the adhesives was measured on a HAAKE RotoVisco 1 rheometer (Waltham, MA, USA) with a parallel plate (P35, 20 mm plate diameter). The distance was 1 mm and testing temperature was 25 °C. The shear rate was obtained in the range of 1 to 120 s^−1^ in 10 s^*−*1^ increments. To compare the apparent viscosities of all the adhesives during the testing, the viscosity value at 1 s^−1^ shear rate was recorded in triplicate and the average values were calculated as the reported values.

### Insoluble Measurements

The residual mass (RM) of the cured adhesives after the hydrolyzation was evaluated in accordance with the procedure described by Luo *et al*.^39^. Briefly, the adhesive specimens were cured in an air-circulating oven at 120 ± 2 °C until reaching a constant weight. The cured adhesives were ground into 100 mesh powder (about 2 g, three replications) and weighed (*m*_1_). The specimens were wrapped with a filter paper and weighted (*m*_0_), then immersed in a beaker with DI water (80 mL) in an oven at 60 ± 2 °C. After 24-h hydrolyzation, the packaged specimens were removed from water and dried to a final constant weight (*m*_2_). The RM of the adhesives was calculated as follows:

3 $$\mathrm{RM}\,( \% )=[1-\frac{{m}_{0}-{m}_{2}}{{m}_{1}}]\times 100$$

where m_0_ and m_1_ are the oven dry weight of adhesive on the conditioning weight (g), m_2_ is the oven dry weight of the sample after hydroxylation.

### Fourier Transform Infrared Spectroscopy (FT-IR)

The adhesive samples were cured and ground into fine (200 mesh) powders for the IR analysis. The IR spectra were recorded on a Nicolet Nexus 6700 spectrometer (Thermo Scientific, Pittsburgh, PA, USA) in the MID-IR range (4000–650 cm^−1^) at a resolution of 4 cm^*−*1^ with 32 scans.

### Solid state ^13^C CP/MAS NMR Spectroscopy (^13^C CP/MAS NMR)

The cured adhesive samples were ground into powder and dried in a vacuum oven (65 °C) for 48 h before the NMR testing. Solid-state ^13^C NMR spectra of the cured adhesive samples were observed under an AVANCE III 400WB spectrometer (Bruker, Switzerland) *et al.* ^13^C frequency of 100.62 MHz using a 4 mm CP/MAS probe. The spinning speed of the samples was set to 14 kHz, contact time of 1 ms, acquisition time of 0.1 s, and recycle delay of 3 s; the spectral width was 300 ppm with 2k data points for each spectrum. The ^13^C NMR chemical shifts were calibrated according to the methine carbon atoms of adamantane (29.47 ppm).

### Thermogravimetric Analysis (TGA)

A thermogravimetric analyzer (TGA Q50: TA instruments, USA) with a temperature range from 25 to 600 °C and a heating rate of 10 °C min^−1^ was used to investigate the thermal stability of cured adhesives in a constant nitrogen atmosphere (100 mL min^−1^).

### X-ray Diffraction Analysis (XRD)

The cured adhesives were ground into powder (200 mesh) for XRD analysis. XRD patterns were measured with a D8 Advance X-ray diffractometer (Bruker AXS, Karlsruhe, Germany) equipped with a Cu Kα radiation source (λ = 0.15405 nm) at 40 kV and 40 mA, and recorded over the region of 2θ from 5 to 40°. The crystallinity of each adhesive sample was calculated in DIFFRAC.EVA V 3.1 software (Bruker, Germany).

### Scanning Electron Microscopy (SEM)

The cured adhesive samples were artificially fractured into several pieces and their fracture surface morphologies were observed under a Hitachi S-3400N SEM (Hitachi Science System, Ibaraki, Japan) after coating the samples with gold.

## References

[1] Han M (2017). 5-Hydroxymethyl-2-vinylfuran: a biomass-based solvent-free adhesive. Green Chem..

[2] Efhamisisi D (2016). Induced Tannin Adhesive by Boric Acid Addition and Its Effect on Bonding Quality and Biological Performance of Poplar Plywood. Acs Sustain. Chem. Eng..

[3] Song, Y., Dai, Z., Wang, Z., Ji, A. & Gorb, S. N. The synergy between the insect-inspired claws and adhesive pads increases the attachment ability on various rough surfaces. *Sci. Rep-Uk*. **6** (2016). 10.1038/srep26219 PMC4873747 27198650

[4] Liu H, Li C, Sun XS (2015). Improved water resistance in undecylenic acid (UA)-modified soy protein isolate (SPI)-based adhesives. Ind. Crop. Prod..

[5] Zhang Y, Zhu W, Lu Y, Gao Z, Gu J (2014). Nano-scale blocking mechanism of MMT and its effects on the properties of polyisocyanate-modified soybean protein adhesive. Ind. Crop. Prod..

[6] Sandberg, D. In *Environmental Impacts of Traditional and Innovative Forest-based Bioproducts* 105–172 (Springer, 2016).

[7] Li X, Luo J, Gao Q, Li J (2016). A sepiolite-based united cross-linked network in a soybean meal-based wood adhesive and its performance. RSC Adv..

[8] Thakur MK, Thakur VK, Gupta RK, Pappu A (2016). Synthesis and Applications of Biodegradable Soy Based Graft Copolymers: A Review. Acs Sustain. Chem. Eng..

[9] Kang H (2016). High-Performance and Fully Renewable Soy Protein Isolate-Based Film from Microcrystalline Cellulose via Bio-Inspired Poly(dopamine) Surface Modification. Acs Sustain. Chem. Eng..

[10] Li J (2015). Soybean meal-based wood adhesive enhanced by ethylene glycol diglycidyl ether and diethylenetriamine. Ind. Crop. Prod..

[11] Serra A (2016). Commercial processed soy-based food product contains glycated and glycoxidated lunasin proteoforms. Sci. Rep-Uk..

[12] Hsia S-Y, Hsiao Y-H, Li W-T, Hsieh J-F (2016). Aggregation of soy protein-isoflavone complexes and gel formation induced by glucono-δ-lactone in soymilk. Sci. Rep-Uk..

[13] Gao Q (2012). Soybean meal‐based adhesive enhanced by MUF resin. J. Appl. Polym. Sci..

[14] Liu Y, Li K (2007). Development and characterization of adhesives from soy protein for bonding wood. Int. J. Adhes. Adhes..

[15] Yuan C, Luo J, Luo J, Gao Q, Li J (2016). A soybean meal-based wood adhesive improved by a diethylene glycol diglycidyl ether: properties and performance. RSC Adv..

[16] Li H, Li C, Gao Q, Zhang S, Li J (2014). Properties of soybean-flour-based adhesives enhanced by attapulgite and glycerol polyglycidyl ether. Ind. Crop. Prod..

[17] Awaja F, Gilbert M, Kelly G, Fox B, Pigram PJ (2009). Adhesion of polymers. Prog. Polym. Sci..

[18] Haeshin Lee, S. M. D., 2 William M. Miller,2,3 Phillip B. Messersmith1,3,4*. Mussel-Inspired Surface Chemistry for Multifunctional Coatings. *Science* **318** (2007). 10.1126/science.1147241 PMC2601629 17947576

[19] Liu Y, Ai K, Lu L (2014). Polydopamine and its derivative materials: synthesis and promising applications in energy, environmental, and biomedical fields. Chem. Rev..

[20] Ye Q, Zhou F, Liu W (2011). Bioinspired catecholic chemistry for surface modification. Chem. Soc. Rev.

[21] Mu Y, Wan X (2016). Simple but Strong: A Mussel-Inspired Hot Curing Adhesive Based on Polyvinyl Alcohol Backbone. Macromol. Rapid. Commun..

[22] Fan C, Fu J, Zhu W, Wang DA (2016). A mussel-inspired double-crosslinked tissue adhesive intended for internal medical use. Acta. Biomater..

[23] Wilchek M, Miron T (2015). Mussel-inspired new approach for polymerization and cross-linking of peptides and proteins containing tyrosines by Fremy’s salt oxidation. Bioconjug. Chem..

[24] Zhong C (2014). Strong underwater adhesives made by self-assembling multi-protein nanofibres. Nat. Nanotechnol..

[25] Ryu JH (2011). Catechol-functionalized chitosan/pluronic hydrogels for tissue adhesives and hemostatic materials. Biomacromolecules.

[26] Lee Y, Lee H, Messersmith PB, Park TG (2010). A Bioinspired Polymeric Template for 1D Assembly of Metallic Nanoparticles, Semiconductor Quantum Dots, and Magnetic Nanoparticles. Macromol. Rapid. Commun..

[27] Liu Y, Li* K (2002). Chemical Modification of Soy Protein for Wood Adhesives. Macromol. Rapid. Commun..

[28] Liu Y, Li K (2004). Modification of Soy Protein for Wood Adhesives using Mussel Protein as a Model: The Influence of a Mercapto Group. Macromol. Rapid. Comm..

[29] Kang SM, Rho J, Choi IS, Messersmith PB, Lee H (2009). Norepinephrine: Material-Independent, Multifunctional Surface Modification Reagent. J. Am. Chem. Soc..

[30] Huang S, Zhang Y, Shi J, Huang W (2016). Superhydrophobic Particles Derived from Nature-Inspired Polyphenol Chemistry for Liquid Marble Formation and Oil Spills Treatment. Acs Sustain. Chem. Eng..

[31] Fan L (2015). Green coating by coordination of tannic acid and iron ions for antioxidant nanofiltration membranes. RSC Adv..

[32] Rahim MA (2014). Coordination-Driven Multistep Assembly of Metal–Polyphenol Films and Capsules. Chem. Mater..

[33] Insaward A, Duangmal K, Mahawanich T (2015). Mechanical, Optical, and Barrier Properties of Soy Protein Film As Affected by Phenolic Acid Addition. J. Agric. Food. Chem..

[34] Kim K (2015). TAPE: A Medical Adhesive Inspired by a Ubiquitous Compound in Plants. Adv. Funct. Mater..

[35] Kang H, Wang Z, Zhang W, Li J, Zhang S (2016). Physico-chemical properties improvement of soy protein isolate films through caffeic acid incorporation and tri-functional aziridine hybridization. Food. Hydrocolloid..

[36] Wu J (2015). Improving the hydrophilicity and fouling resistance of RO membranes by surface immobilization of PVP based on a metal-polyphenol precursor layer. J. Membrane. Sci..

[37] Wang Z, Kang H, Zhang W, Zhang S, Li J (2017). Improvement of interfacial interactions using natural polyphenol-inspired tannic acid-coated nanoclay enhancement of soy protein isolate biofilms. Appl Surf Sci..

[38] Li H (2016). Properties of soybean-flour-based adhesives enhanced by attapulgiteand glycerol polyglycidyl ether. Ind. Crop. Prod..

[39] Luo J (2016). Toughening improvement to a soybean meal-based bioadhesive using an interpenetrating acrylic emulsion network. J. Mater. Sci..

[40] Xu LQ, Pranantyo D, Neoh K-G, Kang E-T, Fu GD (2016). Thiol Reactive Maleimido-Containing Tannic Acid for the Bioinspired Surface Anchoring and Post-Functionalization of Antifouling Coatings. Acs Sustain. Chem. Eng..

[41] Shin M, Kim K, Shim W, Yang JW, Lee H (2016). Tannic Acid as a Degradable Mucoadhesive Compound. ACS Biomater. Sci. Eng..

[42] Chen YN (2016). Poly(vinyl alcohol)-Tannic Acid Hydrogels with Excellent Mechanical Properties and Shape Memory Behaviors. ACS Appl. Mater. Interfaces..

[43] Ma L, Yang Y, Yao J, Shao Z, Chen X (2013). Robust soy protein films obtained by slight chemical modification of polypeptide chains. Polym. Chem..

[44] Preston CM, Trofymow JA (2015). The chemistry of some foliar litters and their sequential proximate analysis fractions. Biogeochemistry.

[45] Zhang XQ (2010). Chemical Cross-Linking Gelatin with Natural Phenolic Compounds as Studied by High-Resolution NMR Spectroscopy. Biomacromolecules.

[46] Dillon EP, Crouse CA, Barron AR (2008). Synthesis, characterization, and carbon dioxide adsorption of covalently attached polyethyleneimine-functionalized single-wall carbon nanotubes. Acs Nano..

[47] Nuryawan A, Singh AP, Zanetti M, Park B-D, Causin V (2017). Insights into the development of crystallinity in liquid urea-formaldehyde resins. Int J Adhes Adhes.

[48] Faris, A. H., Rahim, A. A., Ibrahim, M. N. M., Alkurdi, A. M. & Shah, I. Combination of lignin polyol-tannin adhesives and polyethylenimine for the preparation of green water-resistant adhesives. *J. Appl. Polym. Sci.* **133**, n/a-n/a (2016).

